# Creatine monohydrate modulates testicular morphology and SOD1/NFκB-p65 immunoexpression in streptozotocin-induced diabetic rats

**DOI:** 10.1007/s10735-026-10831-1

**Published:** 2026-05-29

**Authors:** Ludmila Thainá Chaves Freitas, Hailton Pereira de Melo Júnior, Emily Lima Oliveira, Fernanda Carolina Ribeiro Dias, Ruthnaldo Rodrigues Melo de Lima, João Paulo Matos Santos Lima, Matheus Anselmo Medeiros, Flávio Santos da Silva, Karina Carla de Paula Medeiros, Raimundo Fernandes de Araújo Júnior, Danielle Barbosa Morais, Bento João Abreu

**Affiliations:** 1 Department of Morphology, Federal University of Rio Grande Do Norte, Natal, RN Brazil; 2 Department of Natural Sciences, Federal University of São João del Rei, São João del Rei, MG Brazil; 3 Biochemistry and Molecular Biology Graduate Program, Biosciences Center, Federal University of Rio Grande Do Norte, Natal, RN Brazil; 4 Department of Health Sciences, Federal Rural University of the Semi-Arid (UFERSA), Mossoró, RN Brazil; 5 Department of Biological Sciences, Federal University of Ouro Preto, Ouro Preto, MG Brazil

**Keywords:** Testicular toxicity, Inflammation, Spermatogenesis, Oxidative stress, Histomorphometry

## Abstract

Diabetes mellitus (DM) is a chronic metabolic disorder characterized by persistent hyperglycemia that may impair testicular function, including spermatogenesis and steroidogenesis. Creatine, an endogenously synthesized nitrogenous compound and one of the main reservoirs in the testes, is widely used, particularly among men. However, evidence regarding its effects on male reproductive health, especially under DM, remains limited. This study evaluated the impact of creatine supplementation on testicular morphology in streptozotocin-induced diabetic rats. Forty-eight adult Wistar rats were randomly assigned to four groups (n = 12 each): C, control; CT, creatine; D, diabetes; and DT, diabetes + creatine. Creatine-enriched chow was administered in two phases: a loading phase (13%; 130 g/kg) for 5 days prior to DM induction, followed by a maintenance phase (2%; 20 g/kg) for 35 days. Biochemical, histomorphometric, histopathological, and immunohistochemical analyses were performed. Compared to controls, group D showed increased seminiferous tubule and epithelial proportions, epithelial height, tubular volume (TV), and tubulosomatic index (TSI), while intertubular proportions decreased. DT normalized most morphological parameters to C group levels, and attenuated the diabetes-induced increases in TV and TSI. In the intertubular compartment, group D showed increased Leydig cell and connective tissue proportions *versus* C, while DT exhibited larger lymphatic spaces (vs. D) and the greatest Leydig cell nuclear diameter among all groups. Histopathological degeneration was significant in both D and DT and remained higher than in control levels. Immunohistochemistry revealed reduced SOD1 and increased NFκB-p65 expression in DT compared to D. These findings indicate that creatine supplementation is associated with morphometric and histopathological changes in the testicular parenchyma of diabetic rats, along with alterations in oxidative stress and inflammatory markers. Further studies are required to determine the functional implications for male reproductive health.

## Introduction

Diabetes Mellitus (DM) is a chronic metabolic disorder characterized by persistent hyperglycemia resulting from impaired insulin secretion by pancreatic β-cells or reduced insulin sensitivity in peripheral target tissues (Dilworth et al. 2024). According to the International Diabetes Federation Diabetes Atlas (IDF 2025), approximately 589 million adults aged 20–79 years worldwide are currently living with DM, and this number is projected to exceed 850 million by 2050 (IDF 2025).

Chronic hyperglycemia leads to several long-term complications, including microvascular disorders such as retinopathy, nephropathy, and neuropathy, as well as macrovascular events, particularly cardiovascular disease (Zakir et al. 2023). More recently, increasing attention has been directed toward the impact of DM on male reproductive function. Hyperglycemia adversely affects male fertility at multiple levels, impairing seminal quality, the genetic integrity of spermatozoa, spermatogenesis, steroidogenesis, and testosterone production (Wagner et al. 2021). These alterations are largely associated with hyperglycemia-induced oxidative stress, which activates redox-sensitive pathways such as superoxide dismutase (SOD) and nuclear factor kappa-light-chain-enhancer of activated B cells (NFκB-p65), while also promoting inflammatory signaling within the testicular microenvironment (Khalil et al. 2021). These observations underscore the need for therapeutic strategies capable of mitigating DM-related damage, particularly within the male reproductive system.

In this context, creatine monohydrate has emerged as a compound with broad therapeutic potential. Beyond its well-established role in resistance training and sports performance, accumulating evidence indicates that creatine may exert beneficial effects across diverse clinical conditions, including cancer, muscular dystrophies, and neurodegenerative disorders (Solis et al. 2021).

More recently, creatine supplementation has gained attention for its potential relevance in hyperglycemic states, possibly mediated by its metabolic, antioxidant, and cytoprotective properties (Izurieta et al. 2018; Dolan et al. 2019; Gonçalves et al. 2022). Other proposed mechanisms include improved cellular energy metabolism, enhanced glucose uptake, and support of mitochondrial function (Solis et al. 2021). Additionally, in human neuronal and endothelial cells, creatine protects mitochondrial DNA from oxidative damage, thereby contributing to organelle stability and cellular bioenergetic function (Clarke et al. 2020).

Given that physical exercise is an effective strategy for glycemic control, cardiovascular risk reduction, and weight loss in diabetic patients (Pan et al. 2018), and that creatine monohydrate is widely used to enhance performance in this population, its effects on testicular morphology under diabetic conditions remain poorly understood. We hypothesized that creatine supplementation modulates diabetes-induced testicular alterations, potentially influencing structural integrity and cellular composition. Therefore, this study aimed to address this gap by evaluating the effects of creatine monohydrate supplementation on testicular morphology in a streptozotocin (STZ)-induced diabetes model.

## Materials and methods

### Animals and experimental setting

Forty-eight adults male Wistar rats (200–250 g, 90 days old) were obtained from the Animal Facility of the Biosciences Center, Federal University of Rio Grande do Norte (UFRN). Animals were housed in polypropylene cages equipped with standard feeders and water bottles, with up to four rats per cage. Environmental conditions were controlled (24 ± 2 °C; 12 h light/12 h dark cycle), and animals received standard chow and water ad libitum. Body weight was recorded weekly, and water and chow consumption per cage were monitored daily throughout the protocol. All procedures complied with national guidelines for animal experimentation and were approved by the Ethics Committee on Animal Use of UFRN (protocol no. 030.025/2017).

Animals were randomly assigned to four groups: C (control), n = 12; CT (creatine), n = 12; D (DM), n = 12; and DT (DM + creatine), n = 12. All animals were included in biometric, biochemical, histomorphometric, and histopathological analyses.

### Induction of DM

**Fig. 1:**
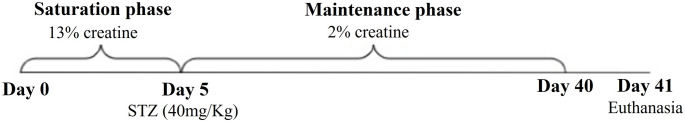
Study design

Following a 14-h fast, animals (except those in the control groups, C and CT), received a single intraperitoneal injection of STZ (40 mg/kg body weight), freshly dissolved in 10 mM sodium citrate buffer (pH 4.5) (Fig. 1). This protocol is widely used to induce an experimental model of type 1 DM through pancreatic β-cell cytotoxicity (Bortolin et al. 2015; Furman 2021; Gonçalves et al. 2022).

After seven days, fasting blood glucose was assessed using a tail-vein blood sample and a portable glucometer (ONETOUCH® ULTRA®). Rats with fasting glycemia > 250 mg/dL were considered diabetic. Glycemia was monitored weekly in all groups, while water and chow consumption were recorded daily. Blood samples were obtained by puncturing the hepatic portal vein for creatinine analysis. After remaining in test tubes for 6 h, the samples were centrifuged (5000 × g) for 15 min to obtain the serum. Creatinine levels were then determined using specific enzymatic colorimetric assays (Labtest, Lagoa Santa, MG, Brazil), according to the manufacturer’s instructions, and absorbance was read with a spectrophotometer.

### Preparation of creatine-enriched chow

Animals in the CT and DT groups received creatine monohydrate supplementation incorporated into an isocaloric chow in two phases (Hultman et al. 1996; Vandenberghe et al. 1997). The loading phase consisted of a 13% creatine-enriched chow (130 g/kg) for 5 days before diabetes induction to promote creatine saturation (Deminice et al. 2009). The maintenance phase began immediately after type 1 DM induction and consisted of a 2% creatine-enriched chow (20 g/kg) supplied for 35 days (Fig. 1). The mixture was homogenized and moistened with 150 mL of water per kg to obtain a firm, cohesive paste (Gonçalves et al. 2022). Animals in the C and D groups received the same isocaloric chow without creatine.

### Biometric parameters

Body weight was individually recorded using a precision scale. Water consumption was calculated by subtracting the residual volume from the total volume replenished daily per cage and normalized per animal. Chow intake was assessed similarly: 150 g of chow were provided daily per cage, and the difference between initial and residual weights was divided by the number of animals to obtain the mean consumption per rat (Gonçalves et al. 2022).

### Euthanasia and tissue collection

At the end of the protocol, animals were euthanized under deep anesthesia induced by isoflurane (3%) inhalation. Loss of pedal and muscular reflexes confirmed deep anesthesia. Thoracotomy was then performed for blood collection and testes were removed.

### Histological analyses

Testes were fixed in 4% paraformaldehyde for 24 h, dehydrated in graded ethanol (70–100%), cleared in xylene, and embedded in paraffin at 60 °C. Semi-serial transverse Sects. (3 µm) were obtained and stained with hematoxylin and eosin (H&E) for histopathological and histomorphometric evaluations.

For each animal, three sections separated by 45 µm were analyzed. Tissue architecture was examined under a light microscope (Leipzig SOLSTICE 4X) coupled to a digital camera at 10× and 40× magnification. Images were analyzed using ImageJ software (version 1.53, NIH, Bethesda, MD, USA), as described elsewhere (Schneider et al. 2012).

### Biometry and histomorphometry of testicular components

The gonadosomatic index (GSI) was calculated using final body weight and the combined weight of both testes, according to Silva et al. (2019):

GSI = (gonadal weight/body weight) × 100.

Ten images per animal (10 × and 40 × objectives) were analyzed using the ImageJ grid tool (80 intersection points per image), following Amann (1970). Points intersecting the seminiferous tubule (basal membrane, epithelium, or lumen) or the intertubular space were quantified. Tubular diameter and seminiferous epithelium height were measured in 20 circular cross-sections per animal, using diametrically opposed measurements (ImageJ, straight-line tool). Tubular volume (TV) was estimated as described by Morais et al. (2017): TV = (gonadal weight × tubular proportion)/100. The tubulosomatic index (TSI) was then calculated: TSI = (TV/body weight) × 100. Histopathological assessment was performed by evaluating 200 randomly selected seminiferous tubules per animal according to the modified Johnsen score (Dias et al. 2023). Under this scale, higher scores represent more severe histopathological alterations. Briefly, score 1: intact seminiferous tubules with germ cells in their normal arrangement and few vacuoles; score 2: vacuoles at the basal portion of the epithelium; score 3: vacuoles at the apical portion of the epithelium; score 4: vacuoles at both basal and apical portions of the epithelium; score 5: spermatogenic cells in the tubular lumen with cellular degeneration; score 6: presence of basal cells only; score 7: presence of Sertoli cells only; and score 8: absence of cells, indicating irreversible degeneration.

### Morphology and morphometry of the intertubular compartment

Ten images per animal (10 × objective) were evaluated using a 26 × 20 grid (520 points per image). Points contacting Leydig cell nuclei or cytoplasm, lymphatic or blood vessels, and connective tissue were recorded. Element percentages were calculated as (absolute count × 100)/total points. The frequency of each element in the testis was calculated as (% intertubule × % element in the intertubule)/100. Element volume was determined relative to the net gonadal weight (gonad weight minus tunica albuginea weight). Total and proportional volumes of each component were then estimated accordingly (Dias et al. 2020, 2025).

### Leydig cell morphometry

Thirty Leydig cell nuclei per animal were measured (ImageJ). The nuclear radius was determined, and nuclear (VNuc), cytoplasmic (VCyto), and cellular (VCell) volumes were calculated as follows: VNuc = 4.19 × 3 × radius; VCyto = (% cytoplasm × VNuc) / % nucleus; VCell = VNuc + VCyto. The total number of Leydig cells per testis was estimated by dividing the total Leydig cell volume by the individual cell volume. The Leydig cell somatic index (LCSI) was calculated as (Leydig cell volume/body weight) × 100 (Dias et al. 2020, 2025).

### Immunohistochemistry (IHC)

IHC was performed according to Oliveira et al. (2021). Three animals per group were randomly selected and analyzed, with three nonconsecutive sections per animal for each marker. Sections were deparaffinized, rehydrated, and subjected to heat-induced antigen retrieval in 0.3% citrate buffer (pH 9.5) at 95 °C, followed by endogenous peroxidase blocking with 3% hydrogen peroxide. Primary antibodies were applied overnight at 4 °C: anti–superoxide dismutase 1 (SOD1; Santa Cruz Biotechnology, Cat# sc-11407, RRID: AB_2193779; 1:200) and anti–NF-κB p65 (Santa Cruz Biotechnology, Cat# sc-8008, RRID: AB_628017; 1:50). Secondary HRP-conjugated antibodies (Thermo Fisher Scientific, Cat# 31466, RRID: AB_10960845; 1:400) were also incubated overnight at 4 °C according to the protocol described by Oliveira et al. (2021).

Immunoreactivity was visualized using diaminobenzidine (DAB; Easypath, Cat# EP-12-20542). Negative controls lacked primary antibodies. Sections were counterstained with hematoxylin and examined under a Nikon E200 LED microscope equipped with a Moticam camera. A semiquantitative scoring system was used to evaluate immunolabeling intensity (IM: 1–4) and extent (EM: 1–5), as described by Cavalcante et al. (2021). The final adjusted score was calculated as (IM × EM)/5.

### Statistical analysis

Data are expressed as mean ± standard deviation (SD). Statistical analyses were performed using GraphPad Prism 9.0 (GraphPad Software, San Diego, USA). One-way ANOVA followed by Tukey’s post-hoc test was used for multiple comparisons. Statistical significance was set at *P* ≤ 0.05.

## Results

### Evaluation of physiologic and biochemical parameters

**Fig. 2:**
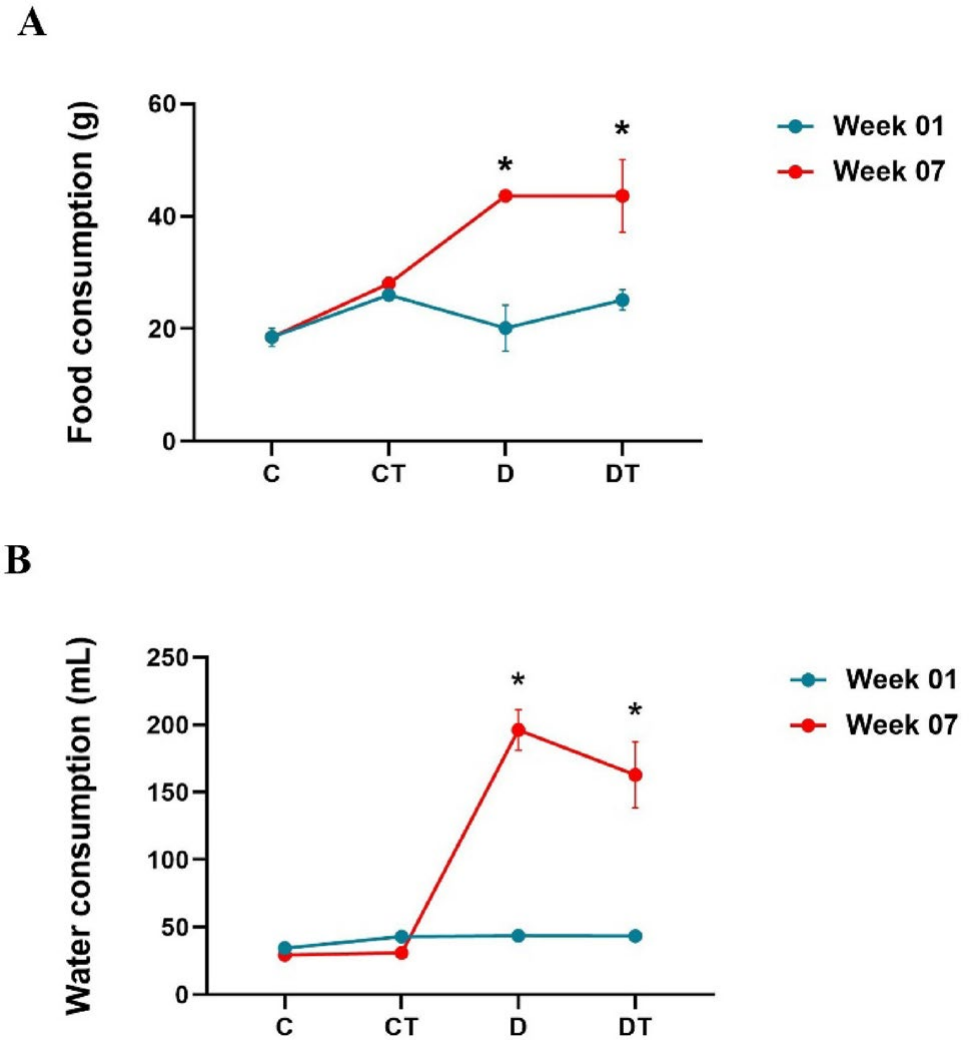
**A** Feed consumption of the animals over the course of 7 weeks. **B** Water consumption of animals over the course of 7 weeks. Experimental groups: control (C), creatine (CT), diabetes (D), and diabetes + creatine (DT). Values are expressed as mean ± standard deviation (SD) with n = 12 animals per group. Differences between groups were analyzed by repeated measures one-way ANOVA, followed by Tukey’s post-hoc test. *P* < 0.001

Repeated-measures analysis revealed a significant main effect of group (*P* < 0.001) and a strong time × treatment interaction. At baseline, all groups showed similar food (Fig. 2A) and water intake (Fig. 2B), with no significant differences among them. However, by the seventh week, the diabetic groups exhibited significant increases compared with their baseline values (Fig. 2A and B).

**Fig. 3:**
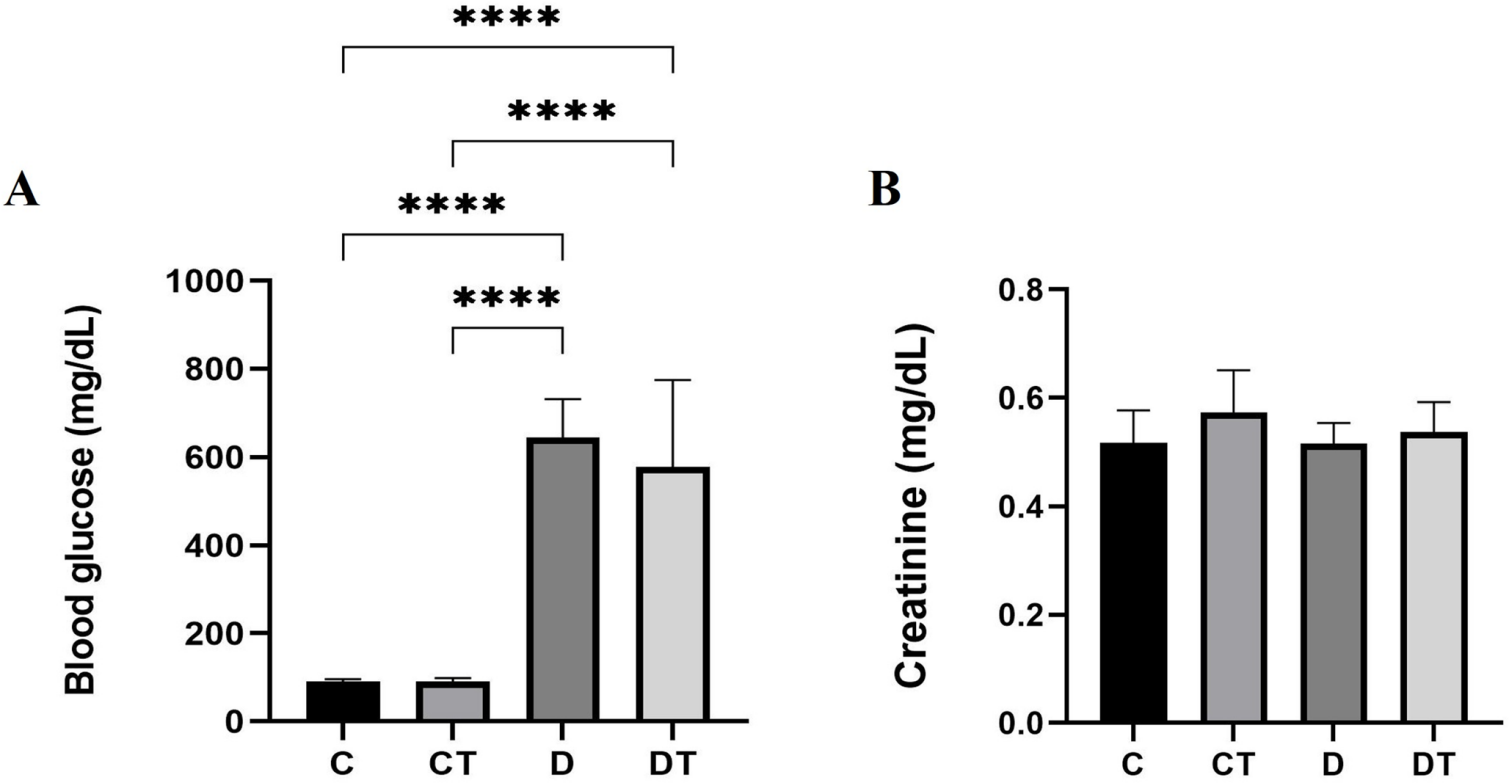
**A** Fasting blood glucose and **B** Serum creatinine of the control (C), creatine (CT), diabetes (D), and diabetes + creatine (DT) groups at the end of the experimental protocol. Values ​​are expressed as mean ± standard deviation (SD) with n = 12 animals per group. Differences between groups were analyzed by one-way ANOVA, followed by Tukey’s post-hoc test. *P < 0.0001

Similar to food and water intake, fasting glucose levels (mg/dL) were significantly higher in the D group compared to the C and CT groups (*P* < 0.0001), confirming the successful induction of DM (Fig. 3A). Creatine supplementation did not significantly alter fasting glucose levels in diabetic animals, as both D and DT groups maintained hyperglycemia relative to controls (Fig. 3B). No statistically significant differences in serum creatinine levels were observed among groups (*P* ≥ 0.05).

### Biometric and histomorphometric parameters

Body weight was significantly reduced in the D group compared to the C and CT groups (*P* < 0.01). Similarly, final body weights in the D group differed significantly from both control groups (Table 1).

**Table 1:** Biometric and histomorphometric parameters of the testes of diabetic rats treated with creatine monohydrate

Morphometric parameters	C (n = 12)	CT (n = 12)	D (n = 12)	DT (n = 12)
Initial body weight (g)	217.50 ± 10.61^d^	218.04 ± 13.96^d^	234.30 ± 18.45	244.90 ± 12.56^ab^
Final body weight (g)	263.00 ± 20.29^ cd^	266.60 ± 12.44^ cd^	193.30 ± 16.51^ab^	200.90 ± 18.51^ab^
Testis weight (g)	2.06 ± 0.15	2.06 ± 0.11	1.82 ± 0.31	1.80 ± 0.21
Seminiferous tubules (%)	67.82 ± 1.55^c^	64.41 ± 2.10^c^	81.13 ± 2.54^abd^	67.84 ± 2.60^c^
Tunica propria (%)	16.91 ± 1.66	16.36 ± 1.09	17.83 ± 1.14	17.18 ± 0.89
Lumem (%)	7.11 ± 1.76	4.69 ± 0.48	5.28 ± 0.70	5.32 ± 0.38
Epithelium (%)	43.80 ± 1.44^c^	43.36 ± 2.60^c^	58.03 ± 2.76^abd^	45.34 ± 2.24^c^
Intertubule (%)	32.18 ± 1.55	35.59 ± 2.10^c^	18.88 ± 2.54^b^	32.16 ± 2.60
Tubular diameter (µm)	260.45 ± 67.42	281.74 ± 10.06	283.35 ± 16.93	278.00 ± 12.12
Epithelial height (µm)	32.00 ± 12.60	25.65 ± 1.78^c^	38.36 ± 1.27^b^	33.16 ± 3.19
TV (mL)	1.40 ± 0.11	1.33 ± 0.06	1.44 ± 0.28^d^	1.22 ± 0.13^c^
GSI (%)	0.84 ± 0.05	0.84 ± 0.05	0.86 ± 0.17	0.82 ± 0.12
TSI (%)	0.57 ± 0.04	0.54 ± 0.04^c^	0.70 ± 0.16^bd^	0.56 ± 0.07^c^

Values are expressed as mean ± standard deviation (SD), with n = 12 animals per group. Experimental groups: Control (C), treated control (CT), diabetic (D), and treated diabetic (DT). GSI, Gonadosomatic index; TSI, Tubulosomatic index; TV, Tubular volume. Differences between groups were analyzed by one-way ANOVA, followed by Tukey’s post-hoc test. Superscript letter indicate significant differences (*P* < 0.05) as follows: “a” versus C; “b” versus CT; “c” versus D; “d” versus DT

Some histomorphometric discrepancies were observed between the C and CT groups. Specifically, CT exhibited a higher proportion of the intertubular area, and a reduced epithelial height compared to C (Table 1; *P* < 0.05).

Diabetic animals showed increased proportions of seminiferous tubules and epithelium, as well as greater epithelial height, tubular volume, and TSI relative to the C and CT groups (Table 1; *P* < 0.05). In contrast, the intertubular proportion was significantly reduced in the D group when compared to controls (Table 1; *P* < 0.05).

Creatine supplementation restored the values of seminiferous tubules, epithelial proportion, intertubular area, and epithelial height to levels comparable to the C group (Table 1; *P* < 0.05). Additionally, the DT group showed reduced TV and TSI compared to the D group (Table 1; *P* < 0.05).

No significant differences were observed among the groups in testis weight, tunica propria, lumen, tubular diameter, or GSI (Table 1; *P* ≥ 0.05).

### Intertubular compartment analyses

Table 2 summarizes the volumetric proportions and absolute volumes of the intertubular compartment components. This compartment was primarily composed of lymphatic spaces, followed by Leydig cells, connective tissue, and blood vessels. Considering the intertubular space, the proportion of Leydig cells was significantly higher in group D compared to groups C and CT (Table 2; *P* < 0.05). Group DT also exhibited higher values than both control groups (Table 2; *P* < 0.05), although these were significantly lower than those observed in group D (Table 2; *P* < 0.05). Blood vessel proportions were significantly higher in group D compared to groups CT and DT (Table 2; *P* < 0.01), whereas lymphatic spaces were decreased in group D relative to the other groups (Table 2; *P* < 0.05). Interestingly, groups CT and DT exhibited a reduced proportion of connective tissue compared to groups C, and groups C and D, respectively (Table 2; *P* < 0.05).

**Table 2:** Volumetric proportion and volume of the components of the intertubular compartment of the testes in diabetic rats treated with creatine monohydrate

Parameters	C (n = 12)	CT (n = 12)	D (n = 12)	DT (n = 12)
Percentage in the intertubular space (%)				
Leydig cells	3.42 ± 1.06^c^	4.21 ± 0.81^c^	9.49 ± 2.12^abd^	6.75 ± 1.37^abc^
Blood vessels	1.688 ± 1.30	1.019 ± 0.49^c^	2.155 ± 0.64^bd^	0.686 ± 0.34^c^
Lymphatic spaces	90.4 ± 2.33^c^	92.26 ± 1.26^c^	83.4 ± 3.30^abd^	90.23 ± 1.83^c^
Connective tissue	4.47 ± 1.00^bd^	2.52 ± 0.53^ac^	4.96 ± 0.85^bd^	2.34 ± 0.77^ac^
Testicular volume (mL)				
Leydig cells	0.02 ± 0.01^d^	0.03 ± 0.01	0.03 ± 0.01	0.04 ± 0.01^a^
Blood vessels	0.012 ± 0.01	0.008 ± 0.01	0.008 ± 0.01	0.004 ± 0.00
Lymphatic spaces	0.60 ± 0.05^c^	0.68 ± 0.07^ cd^	0.28 ± 0.03^abd^	0.52 ± 0.08^bc^
Connective tissue	0.03 ± 0.01^bcd^	0.02 ± 0.00^a^	0.02 ± 0.00^a^	0.010 ± 0.00^a^
Percentage in the testicular tissue (%)				
Leydig cells	1.10 ± 0.35^ cd^	1.49 ± 0.26^d^	1.78 ± 0.31^a^	2.17 ± 0.52^ab^
Blood vessels	0.544 ± 0.42	0.361 ± 0.17	0.401 ± 0.10	0.223 ± 0.12
Lymphatic spaces	29.11 ± 1.80^bc^	32.85 ± 2.25^acd^	15.95 ± 2.55^abd^	28.95 ± 2.26^bc^
Connective tissue	1.43 ± 0.25^bcd^	0.89 ± 0.18^a^	0.84 ± 0.18^a^	0.75 ± 0.25^a^

Values are expressed as mean ± standard deviation (SD), with n = 12 animals per group. Experimental groups: Control (C), treated control (CT), diabetic (D), and treated diabetic (DT). Differences between groups were analyzed by one-way ANOVA, followed by Tukey’s post-hoc test. Superscript letter indicate significant differences (*P* < 0.05) as follows: “a” versus C; “b” versus CT; “c” versus D; “d” versus DT

In addition, the volume of Leydig cells per testis was significantly higher in the DT group compared with the control (Table 2; *P* < 0.05). The D group exhibited smaller lymphatic spaces than the C and CT groups, while DT promoted an increase in these spaces to control levels. DT also exhibited higher lymphatic spaces when compared to CT and D in the testis volume (Table 2; *P* < 0.05).

Regarding the proportion of Leydig cells within the testis, the D and DT groups showed a greater number of cells than the C group; furthermore, the DT group was significantly higher than the CT group (Table 2; *P* < 0.05). Moreover, the lymphatic spaces, which were increased in CT and decreased in D, were restored to control levels in the DT group (Table 2; *P* < 0.05). In contrast, the CT, D, and DT groups demonstrated a lower percentage of connective tissue compared with the C group (Table 2; *P* < 0.05).

### Leydig cells morphometry

Table 3 presents the morphometric parameters of Leydig cells. Nuclear diameter was significantly increased in the DT group compared with the D group (*P* < 0.05). Cytoplasmic volume was increased in the DT group compared with the D and C groups (Table 3; *P* < 0.05). The number of Leydig cells per gram of testis was higher in the diabetic animals compared with the control group (Table 3; *P* < 0.05). The LCI was significantly increased in the DT group compared with the control group (Table 3; *P* < 0.05). No significant difference was observed among groups for the remaining parameters, such as nuclear percentage, nuclear volume, cytoplasmic percentage, Leydig cell volume, and the number of Leydig cells per testis (Table 3, *P* > 0.05).

**Table 3:** Histomorphometry of Leydig cells from the testes of diabetic rats treated with creatine monohydrate

Morphometric parameters of Leydig cells	C (n = 12)	CT (n = 12)	D (n = 12)	DT (n = 12)
Nuclear diameter (µm)	5.25 ± 0.17	5.28 ± 0.34	4.92 ± 0.24^d^	5.35 ± 0.24^c^
Nuclear percentage (%)	33.65 ± 8.80	28.06 ± 4.71	25.87 ± 4.22	23.87 ± 6.85
Nuclear volume (µm)	76.19 ± 7.47	77.99 ± 15.49	62.73 ± 9.25	80.82 ± 11.05
Cytoplasmic percentage (%)	66.35 ± 8.80^d^	71.94 ± 4.70	74.13 ± 4.22	76.13 ± 6.85^a^
Cytoplasmic volume (µm^3^)	163.30 ± 59.96^d^	210.47 ± 79.59	182.77 ± 37.09	280.60 ± 105.98^a^
Leydig cell volume (µm^3^)	239.49 ± 62.34	288.47 ± 92.83	245.5 ± 4.22	361.42 ± 105.85
Number of Leydig cells per testis (× 10⁶)	95.45 ± 23.36	118.00 ± 49.08	131.33 ± 19.70	110.70 ± 21.60
Number of Leydig cells per gram of testis (× 10^5^)	46.04 ± 9.67^c^	57.21 ± 23.23	72.65 ± 6.58^a^	62.56 ± 16.05
LCSI (%)	0.009 ± 0.00^d^	0.013 ± 0.00	0.015 ± 0.00	0.018 ± 0.00^a^

Values are expressed as mean ± standard deviation (SD), with n = 12 animals per group. Experimental groups: Control (C), treated control (CT), diabetic (D), and treated diabetic (DT). LCSI, Leydig cell somatic index. Differences between groups were analyzed by one-way ANOVA, followed by Tukey’s post-hoc test. Superscript letter indicate significant differences (*P* < 0.05) as follows: “a” versus C; “b” versus CT; “c” versus D; “d” versus DT

### Histopathology

**Fig. 4:**
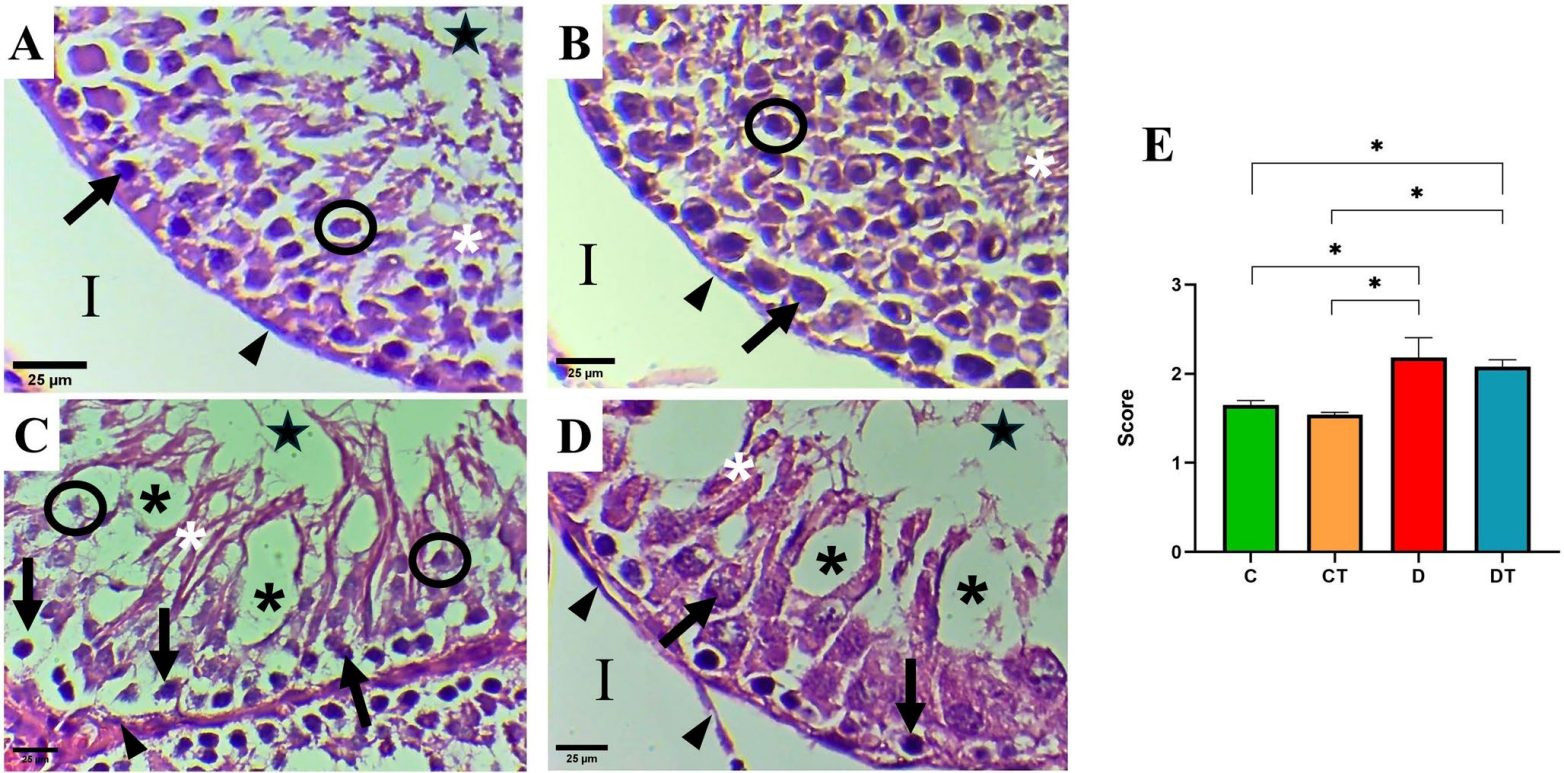
Histopathological analysis using the Johnsen score. **A** Control (C); **B** creatine (CT); **C** diabetes (D); **D** Diabetes + creatine (DT) (Hematoxylin Eosin staining × 400 magnification) and **E** Graphical representation of the mean Johnsen scores for each experimental group. Arrow: primary spermatocytes. Circle: round spermatids. White asterisk: elongated spermatids. Black asterisk: vacuoles. Arrowhead: tunica propria of seminiferous tubule. Star: lumen of seminiferous tubules. I: intertubule. Data are expressed as mean ± standard deviation (SD), with n = 12 animals per group. Differences between groups were analyzed by one-way ANOVA, followed by Tukey’s post-hoc test. *P < 0.0001

The C and CT groups exhibited well-preserved seminiferous tubules with only minor alterations, such as occasional vacuoles in the basal and apical regions of the epithelium (Figs. 4A and B). No relevant morphological differences were observed between these two control groups. The organization of the seminiferous tubules in groups D and DT showed significant alterations. In group D, frequent vacuolization was observed in both the basal and apical regions of the seminiferous epithelium, differing significantly from both control groups (*P* < 0.05; Fig. 4). Of particular note were the pyknotic appearance of the primary spermatocytes and the overall desquamated state of the epithelium (Fig. 4C). Although the seminiferous tubules in group DT also exhibited vacuolization that differed significantly from the control groups (*P* < 0.05), the nuclei of primary spermatocytes (especially those located in the middle region of the epithelium) appeared better preserved than in group D. Additionally, less germ cell desquamation was observed in this group (Fig. 4D). The tunica propria also showed subtle histoarchitectural changes in groups D and DT, characterized by a slightly irregular contour and mild desquamation of myoid cells (Fig. 4C and D).

Regarding histopathological damage, D group exhibited significantly higher Johnsen scores compared to controls (*P* < 0.0001; Fig. 4E). Notably, creatine supplementation failed to attenuate this damage, as scores in the DT group remained elevated and did not return to control levels.

### IHC

**Fig. 5:**
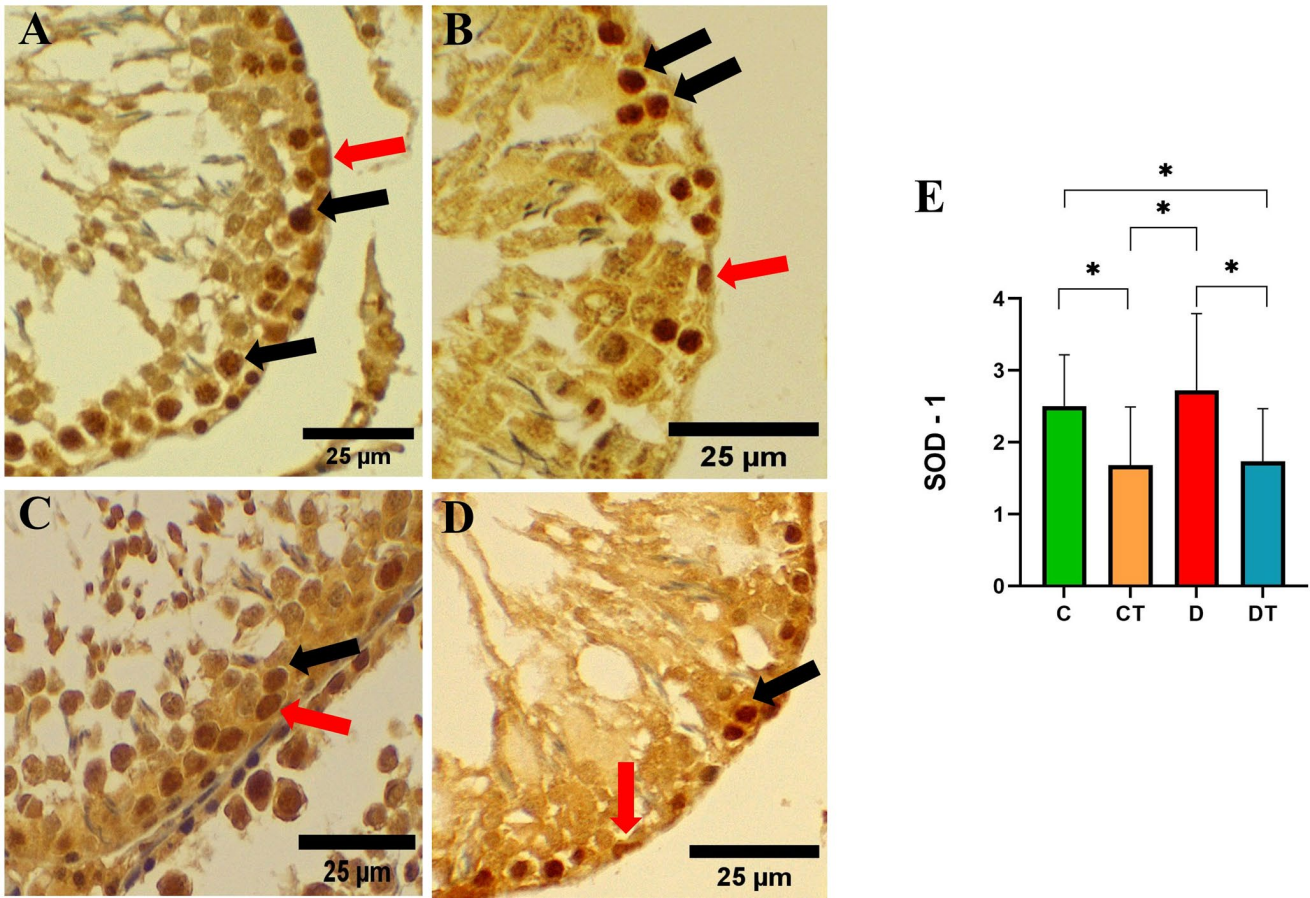
Immunostaining of the antioxidant enzyme SOD1 (superoxide dismutase 1) in the testicular parenchyma of the different experimental groups, evaluated by immunohistochemical technique. **A** Control (C); **B** creatine (CT); **C** Diabetes (D); **D** Diabetes + creatine (DT) (Hematoxylin & DAB staining, 400% magnification) and **E** Graphical representation of the semi-quantitative score of the SOD1 immunostaining, considering the intensity and extent of tissue staining. Black arrow: primary spermatocytes. Red arrow: spermatogonia. Values are expressed as mean ± standard deviation (SD), with n = 3 animals per group. Differences between groups were analyzed by one-way ANOVA, followed by Tukey’s post-hoc test. *P < 0.0001

Semiquantitative scoring revealed that SOD1 immunolabeling levels were similar between the C and D groups (Fig. 5A–E; *P* > 0.05). Notably, creatine supplementation significantly reduced SOD1 immunoexpression in both the CT and DT groups compared to their respective non-supplemented controls (Fig. 5A–E; *P* < 0.0001).

**Fig. 6:**
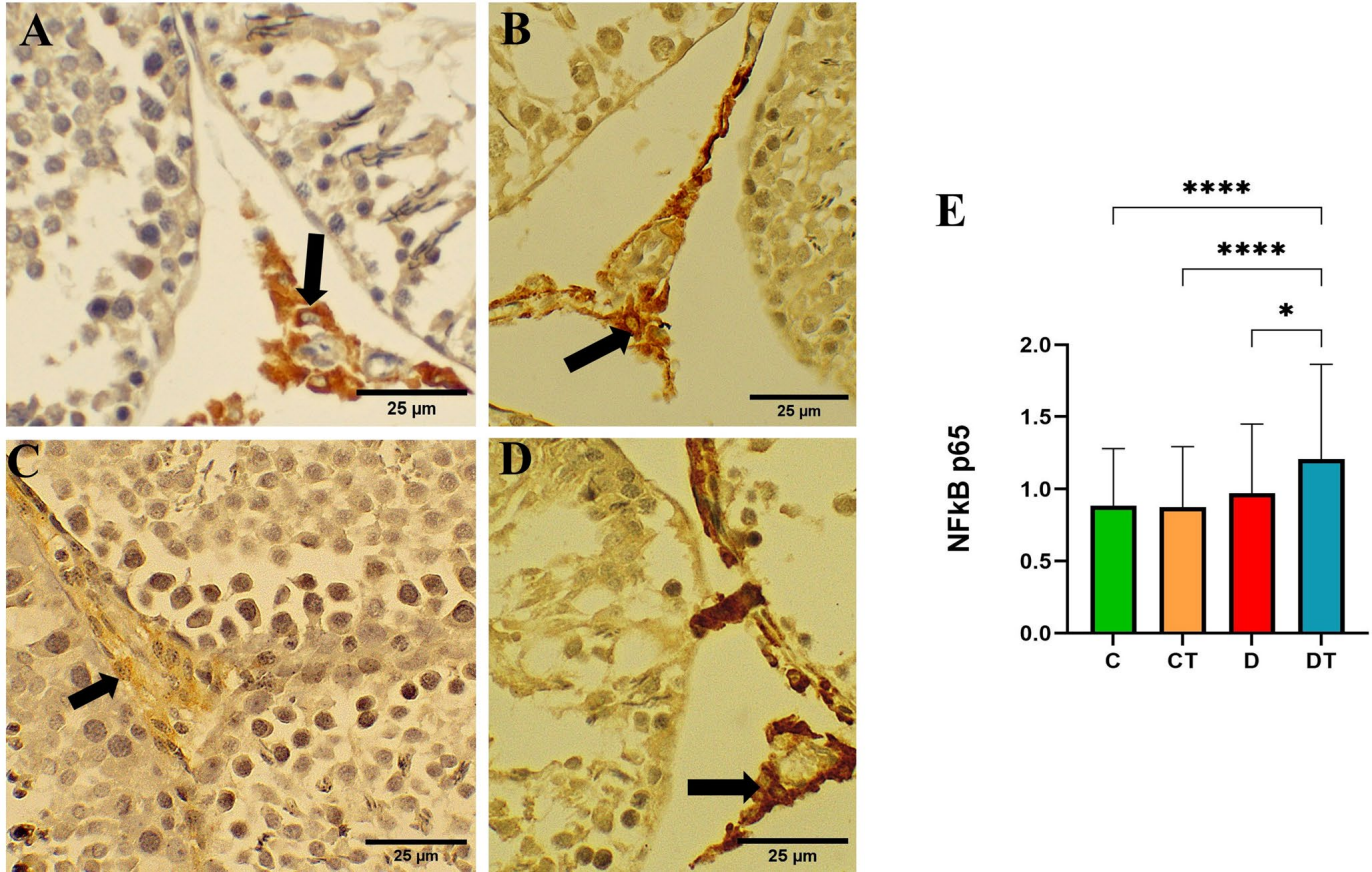
NFκB-p65 immunolabeling in the testicular parenchyma of the different experimental groups, evaluated by immunohistochemical technique. **A** Control (C); **B** creatine (CT); **C** Diabetes (D); **D** Diabetes + creatine (DT) (Hematoxylin & DAB staining, 400% magnification) and **E** Graphical representation of the semi-quantitative score of immunostaining for NFκB p65, considering the intensity and extent of tissue staining. Arrow: Leydig cell. Values are expressed as mean ± standard deviation (SD), with n = 3 animals per group. Differences between groups were analyzed by one-way ANOVA, followed by Tukey’s post-hoc test. *P < 0.05 and ***P < 0.0001

Semiquantitative analysis of NFκB-p65 immunolabeling revealed comparable intensities among the C, CT, and D groups (Fig. 6A–E; *P* > 0.05). In contrast, the DT group exhibited significantly higher expression levels relative to all other groups, as confirmed by the semiquantitative scoring (Fig. 6A–E; *P* ≤ 0.05).

## Discussion

In this study, creatine supplementation did not alter glycemia in diabetic animals but significantly modulated testicular morphology. It reduced epithelial and intertubular parameters observed in DM, including tubular volume, somatic index, and Leydig cell proportion, while expanding lymphatic spaces and increasing Leydig cell nuclear diameter. Creatine also altered connective tissue and lymphatic space parameters in the CT group relative to controls. Despite these changes, supplementation failed to mitigate testicular histopathology in diabetic animals and was associated with reduced SOD1 expression and increased NFκB-p65 immunolabeling. These results suggest persistent oxidative and inflammatory signaling and highlight that creatine modulates the intertubular compartment even in the absence of diabetes.

To date, studies evaluating the effects of creatine monohydrate on testicular integrity in diabetic models remain scarce, a gap that underscores the rationale for the present investigation. This study suggests that while creatine supplementation does not fully prevent diabetes-induced testicular damage, it significantly modulates morphological and cellular responses to metabolic stress.

Hyperglycemia typically induces structural damage to the seminiferous epithelium and Sertoli cells (Mogaddami et al. 2018). Contrary to these expectations, we observed an increased proportion of seminiferous tubules in diabetic animals. This finding may reflect a compensatory expansion of the testicular niche in response to diabetes-induced injury. Under metabolic stress, Sertoli cells often undergo reprogramming that disrupts the lactate supply essential for spermatogenesis (Alves et al. 2013). This instability may trigger a cellular survival mechanism where depleted germ cell populations increase their mitotic rate to maintain epithelial integrity (Pohl et al. 2021). Thus, while diabetes-induced metabolic shifts cause oxidative stress, they may also stimulate transient germ cell proliferation to replace impaired cells, demonstrating a degree of resilience in the testicular environment despite significant metabolic derangement (Alves et al. 2013).

Moreover, group D exhibited increases in epithelial height, tubular volume, and the tubular somatic index compared to group CT. These results contrast with reports of testicular atrophy and germ cell loss typically associated with STZ-induced diabetes (Oksanen 1975; Shahat et al. 2018). In this model, the observed epithelial expansion indicates a structural rearrangement in response to metabolic stress. However, this morphological change does not correlate with improved spermatogenic function, suggesting instead a transient maintenance of tubular architecture that precedes functional decay.

In this sense, the preservation of tubular proportion in the DT group indicates that creatine supplementation partially maintains the structural organization of the seminiferous compartment, although this does not translate into functional recovery. Regarding the intertubular compartment, group D exhibited a higher proportion of Leydig cells (both in total volume and percentage) compared to groups C and CT. This expansion reflects Leydig cell hypertrophy or hyperplasia, a morphological remodeling consistent with the androgenic dysfunction often observed in diabetic models (Fielitz et al. 2025).

The increased proportion of connective tissue in group D aligns with previous reports of intertubular expansion under diabetic conditions (Ismail et al. 2017), reflecting early remodeling of the testicular niche in response to hyperglycemic stress. While the intertubular compartment is inherently rich in collagen types I, III, and IV, the observed expansion suggests a quantitative shift in these structural components. Additionally, the reduction of lymphatic space in diabetic animals is consistent with impaired intertubular organization and remodeling (Long et al. 2018). Creatine supplementation (DT group) increased the lymphatic space proportion compared to untreated diabetic animals, indicating a modulation of intertubular architecture, likely secondary to changes in other compartment components rather than a direct lymphangiogenic effect.

The percentage of Leydig cells, along with their nuclear diameter and cytoplasmic volume, was increased in the DT group compared to groups C and CT. These morphological changes indicate Leydig cell hypertrophy or hyperplasia, likely resulting from diabetes-induced remodeling or creatine-mediated metabolic effects. While nuclear enlargement is often associated with steroidogenic activity, in this context it may reflect an adaptive response to metabolic stress rather than increased hormonal output, particularly since the testes represent a significant creatine reservoir for ATP production (Kuribayashi et al. 2024). Furthermore, considering the energy demands of the intertubular compartment, these alterations are consistent with the hyperplastic responses reported in other tissues following creatine supplementation (Burke et al. 2023). However, in the absence of endocrine data, these structural shifts do not establish a recovery of steroidogenic function.

While creatine provides bioenergetic support, Leydig cell function is primarily regulated by the hypothalamic–pituitary–gonadal axis (Burke et al. 2023). Consequently, increased intratesticular creatine availability does not inherently result in enhanced testosterone production (Zirkin et al. 2018). In this context, the Leydig cell nuclear enlargement observed in the DT group is more accurately characterized as an adaptive morphological response to metabolic stress rather than an indicator of functional improvement. These structural shifts highlight the selective influence of creatine on testicular architecture, which occurs independently of systemic hormonal recovery.

Groups D and DT exhibited higher histopathological scores than controls, confirming that creatine supplementation does not mitigate diabetes-induced seminiferous damage. The observed epithelial vacuolization, germ cell loss, and structural disorganization align with classical reports of progressive germinal degeneration under chronic hyperglycemia (Oksanen 1975; Rossi et al. 1982) and recent findings of impaired sperm parameters in diabetic models (Zhang et al. 2025)

Although the testes are a known creatine reservoir, its specific physiological role in this tissue remains unclear. While peripheral conversion of dihydrotestosterone may increase with supplementation (Van der Merwe et al. 2009), direct influence on testicular synthesis is not established. Consequently, although reduced intratesticular creatine is associated with infertility (Kuribayashi et al. 2024), our results demonstrate that exogenous supplementation is insufficient to maintain epithelial integrity under diabetic stress.

Diabetes disrupts redox homeostasis by increasing reactive oxygen species (ROS) and weakening antioxidant defenses (Shrilatha and Muralidhara 2007). In this work, untreated diabetic rats exhibited increased SOD1 immunolabeling, likely representing a compensatory response to heightened oxidative stress, given SOD’s role as a primary antioxidant defense (Jomova et al. 2024). Furthermore, SOD1-derived H_2_O_2_ functions as a redox signaling molecule in various cellular pathways (Fukai and Ushio-Fukai 2011). Conversely, the reduced SOD1 labeling in groups CT and DT indicates a distinct redox state. This lower expression suggests either a reduced oxidative demand or a late-stage depletion of antioxidant capacity, as the 40-day experimental period may have bypassed the peak of early redox fluctuations typical of initial oxidative imbalance.

SOD1 activity is linked to NFκB-p65 signaling, as SOD-derived H_2_O_2_ can promote pathway activation (Fukai and Ushio-Fukai 2011). In this study, NFκB-p65 immunolabeling was markedly increased only in the DT group, indicating that the pro-inflammatory effects of creatine are context-dependent and manifest specifically under metabolic compromise. This observation aligns with reports of creatine-induced inflammatory modulation in other disease models. Importantly, the increased NFκB-p65 labeling reflects pathway activation rather than a definitive increase in inflammatory output. These results demonstrate that creatine modulates NFκB-p65-dependent signaling in the diabetic testis, underscoring the importance of investigating downstream mediators such as IL-6 and TNF-α to further characterize this response.

This study has limitations that warrant acknowledgment. The 40-day experimental period does not encompass the complete spermatogenic cycle, and hormonal profiles were not assessed. Additionally, although the IHC analyses were conducted on a relatively small sample size, it was statistically sufficient to detect biologically meaningful differences (Taylor and Levenson 2006). Future research incorporating comprehensive endocrine, molecular, and functional reproductive assessments is essential to determine whether these creatine-induced morphological changes translate into significant impacts on testicular steroidogenesis and fertility.

## Conclusions

Creatine monohydrate supplementation was associated with significant morphometric and histopathological changes in the testes of diabetic rats, specifically modulating the testicular parenchyma and Leydig cell architecture. These findings demonstrate that creatine alters testicular tissue organization under diabetic conditions without providing definitive protection against diabetes-induced damage. Furthermore, the reduction in SOD1 and the concomitant increase in NF-κB p65 immunoexpression indicate the persistence of oxidative and inflammatory signaling. 

Overall, creatine exerts context-dependent effects on testicular morphology in the presence of DM. Given its widespread use, future studies incorporating endocrine and functional reproductive analyses are essential to fully elucidate the impact of creatine on male fertility.

## Data Availability

All data supporting the findings of this study are available within the article. Further enquiries can be directed to the corresponding author.

## References

[CR2] Alves MG, Martins AD, Cavaco JE, Socorro S, Oliveira PF (2013) Diabetes, insulin-mediated glucose metabolism and Sertoli/blood-testis barrier function. Tissue Barriers 1:e23992. 10.4161/tisb.23992 24665384 10.4161/tisb.23992 PMC3875609

[CR3] Amann RP (1970) Sperm production rates. In: Johnson AD, Gomes WR, Vandemark NL (eds). The Testis. Volume 1. New York: Academic Press, pp 433–482. 10.12691/wjar-7-4-6

[CR8] Bortolin RH, da Graça Azevedo Abreu BJ, Abbott Galvão Ururahy M, Costa de Souza KS, Bezerra JF, Loureiro MB, da Silva FS, Marques DE, Batista AA, Oliveira G, Luchessi AD, Lima VM, Miranda CE, Lia Fook MV, Almeida M, de Rezende LA, de Rezende AA (2015) Protection against T1DM-induced bone loss by zinc supplementation: biomechanical, histomorphometric, and molecular analyses in STZ-induced diabetic rats. PLoS ONE 10:e0125349. 10.1371/journal.pone.0125349 25933189 10.1371/journal.pone.0125349 PMC4416905

[CR9] Burke R, Pinero A, Coleman M, Mohan A, Sapuppo M, Augustin F, Aragon AA, Gandow DG, Forbes SC, Swinton P, Schoenfeld BJ (2023) The effects of creatine supplementation combined with resistance training on regional measures of muscle hypertrophy: A systematic review with meta-analysis. Nutrients 15(9):2116. 10.3390/nu15092116 37432300 10.3390/nu15092116 PMC10180745

[CR10] Cavalcante RS, Ishikawa U, Silva ES, Silva-Júnior AA, Araújo AA, Cruz LJ, Chan AB, Júnior RFDA (2021) STAT3/NF-κB signalling disruption in M2 tumour-associated macrophages is a major target of PLGA nanocarriers/PD-L1 antibody immunomodulatory therapy in breast cancer. Br J Pharmacol 178(11):2284–2304. 10.1111/bph.15373 33434950 10.1111/bph.15373 PMC8251773

[CR12] Clarke H, Kim DH, Meza CA, Ormsbee MJ, Hickner RC (2020) The evolving applications of creatine supplementation: could creatine improve vascular health? Nutrients 12(9):2834. 10.3390/nu12092834 32947909 10.3390/nu12092834 PMC7551337

[CR13] Deminice R, Porteri GV, Vannuchi H, Jordão AF (2009) Effects of creatine supplementation on homocysteine levels and lipid peroxidation in rats. Br J Nutr 102(1):110. 10.1017/S0007114508162985 19079843 10.1017/S0007114508162985

[CR16] Dias FC, Matta SLP, Otoni WC, Gomes ML (2025) *Pfaffia glomerata* tetraploid extract induces penile erection and modulates Leydig cell hormonal production. Pharmacol Res Nat Prod. 10.1016/j.prenap.2025.100326

[CR14] Dias FCR, Gomes MLM, Melo FCSA, Menezes TP, Martins AL, Cupertino MC, Otoni WC, da Matta SLP (2020) *Pfaffia glomerata* hydroalcoholic extract stimulates penile tissue in adult Swiss mice. J Ethnopharmacol 261:113182. 10.1016/j.jep.2020.113182 32730872 10.1016/j.jep.2020.113182

[CR15] Dias FCR, Matta SLP, Lima GDA, Souza ACF, Menezes TP, Melo FCSA, Otoni WC, Neves MM, Gomes MLM (2023) *Pfaffia glomerata* polyploid accession compromises male fertility and fetal development. J Ethnopharmacol 314:116680. 10.1016/j.jep.2023.116680 37230282 10.1016/j.jep.2023.116680

[CR17] Dilworth L, Stennett D, Facey A, Omoruyi F, Mohansingh S, Omoruyi FO (2024) Diabetes and the associated complications: the role of antioxidants in diabetes therapy and care. Biomed Pharmacother 181:117641. 10.1016/j.biopha.2024.117641 39541789 10.1016/j.biopha.2024.117641

[CR18] Dolan E, Gualano B, Rawson ES (2019) Beyond muscle: the effects of creatine supplementation on brain creatine, cognitive processing, and traumatic brain injury. Eur J Sport Sci 19(1):1–14. 10.1080/17461391.2018.1500644 30086660 10.1080/17461391.2018.1500644

[CR19] Frielitz-Wagner IV, Klöting N, Kulle A, Rieck K, Söder O, Hiort O (2025) Diabetes type 1 can induce testicular atrophy with Leydig cell hyperplasia and germ cell depletion and therefore negatively influence reproductive function in rats. Endocr Abstr 110:P8. 10.1530/endoabs.110.P8

[CR20] Fukai T, Ushio-Fukai M (2011) Superoxide dismutases: role in redox signaling, vascular function, and diseases. Antioxid Redox Signal 15:1583–1606. 10.1089/ars.2011.3999 21473702 10.1089/ars.2011.3999 PMC3151424

[CR21] Furman BL (2021) Streptozotocin-induced diabetic models in mice and rats. Curr Protoc 1(4):e78. 10.1002/0471141755.ph0547s70 33905609 10.1002/cpz1.78

[CR22] Gonçalves MG, Medeiros MA, de Lemos LIC, de Fátima Campos Pedrosa L, de Andrade Santos PP, Abreu BJ, Lima JPMS (2022) Effects of creatine supplementation on histopathological and biochemical parameters in the kidney and pancreas of streptozotocin-induced diabetic rats. Nutrients 14(3):431. 10.3390/nu14030431 35276790 10.3390/nu14030431 PMC8840440

[CR25] Hultman E, Söderlund K, Timmons JA, Cederblad G, Greenhaff PL (1996) Muscle creatine loading in men. J Appl Physiol 81(1):232–237. 10.1152/jappl.1996.81.1.232 8828669 10.1152/jappl.1996.81.1.232

[CR26] IDF Diabetes Atlas [Internet]. 11th ed (2025) 130 p. Disponível em: https://diabetesatlas.org/media/uploads/sites/3/2025/04/IDF_Atlas_11th_Edition_2025.pdf

[CR27] Ismail SA, Omoregie E, Al-Hadi D, Alshahrani A, Hadeed A (2017) Impaired expression of testicular androgen receptor and collagen fibers in the testis of diabetic rats under HAART: the role of *Hypoxis hemerocallidea*. Folia Histochem Cytobiol 55(2):101–112. 10.5603/FHC.a2017.0016 10.5603/FHC.a2017.0016 28994096

[CR28] Izurieta Munoz H, Gonzales EB, Sumien N (2018) Effects of creatine supplementation on nociception in young male and female mice. Pharmacol Rep 70(2):316–321. 10.1016/j.pharep.2017.11.002 29477040 10.1016/j.pharep.2017.11.002 PMC5882535

[CR30] Jomova K, Alomar SY, Alwasel SH, Nepovimova E, Kuca K, Valko M (2024) Several lines of antioxidant defense against oxidative stress: antioxidant enzymes, nanomaterials with multiple enzyme-mimicking activities, and low-molecular weight antioxidants. Arch Toxicol 98:1323–1367. 10.1007/s00204-024-03696-4 38483584 10.1007/s00204-024-03696-4 PMC11303474

[CR31] Khalil ASM, Giribabu N, Yelumalai S, Shahzad H, Kilari EK, Salleh N (2021) Myristic acid defends against testicular oxidative stress, inflammation, apoptosis: restoration of spermatogenesis, steroidogenesis in diabetic rats. Life Sci 278:119605. 10.1016/j.lfs.2021.119605 33989665 10.1016/j.lfs.2021.119605

[CR33] Kuribayashi S, Fukuhara S, Kitakaze H, Tsujimura G, Imanaka T, Ueda N, Takezawa K, Ikawa M, Nonomura N (2024) Intratesticular creatine maintains spermatogenesis by defining tight junctions. Sci Rep 14:30692. 10.1038/s41598-024-77986-3 39730393 10.1038/s41598-024-77986-3 PMC11680987

[CR34] Long L, Liang S, Lin J, Wang Y, Hu H, Zhou X (2018) Hyperglycemia induced testicular damage in type 2 diabetes mellitus rats exhibiting microcirculation impairments associated with vascular endothelial growth factor decreased via PI3K/Akt pathway. Oncotarget 9(4):5321–5336. 10.18632/oncotarget.23915 29435181 10.18632/oncotarget.23915 PMC5797052

[CR35] Mogaddami Z, Sheikhzadeh F, Hatami H, Khojasteh SMB, Khajehnasiri N, Hemmati ARA, Dastranj A (2018) Effects of short- and long-term regular exercise on reproductive tissue in streptozotocin-induced diabetic male Wistar rats. Endocr Regul 52(4):167–175. 10.2478/enr-2018-0021 31517613 10.2478/enr-2018-0021

[CR36] Morais DB, Puga LC, Paula TA, Freitas MB, Matta SL (2017) The spermatogenic process of the common vampire bat *Desmodus rotundus* under a histomorphometric view. PLoS ONE 12(3):e0173856. 10.1371/journal.pone.0173856 28301534 10.1371/journal.pone.0173856 PMC5354406

[CR38] Oksanen A (1975) Testicular lesions of streptozotocin diabetic rats. Horm Res 6:138–144. 10.1159/000178671 130334 10.1159/000178671

[CR39] Oliveira ALCDSL, Zerillo L, Cruz LJ, Schomann T, Chan AB, De Carvalho TG, Souza SVP, Araújo AA, de Geus-Oei LF, Júnior RFDA (2021) Maximizing the potency of oxaliplatin coated nanoparticles with folic acid for modulating tumor progression in colorectal cancer. Mater Sci Eng C Mater Biol Appl 120:111678. 10.1016/j.msec.2020.111678 33545840 10.1016/j.msec.2020.111678

[CR40] Pan B, Ge L, Xun YQ, Chen YJ, Gao CY, Han X, Zuo LQ, Shan HQ, Yang KH, Ding GW, Tian JH (2018) Exercise training modalities in patients with type 2 diabetes mellitus: a systematic review and network meta-analysis. Int J Behav Nutr Phys Act 15(1):72. 10.1186/s12966-018-0703-3 30045740 10.1186/s12966-018-0703-3 PMC6060544

[CR42] Pohl E, Gromoll J, Wistuba J, Laurentino S (2021) Healthy ageing and spermatogenesis. Reproduction 161:R89–R101. 10.1530/REP-20-0633 33574214 10.1530/REP-20-0633

[CR44] Rossi GI, Aeschlimann M (1982) Morphometric studies of pituitary glands and testes in rats with streptozotocin–induced diabetes. Andrologia 14:532–542. 10.1111/j.1439-0272.1982.tb02306.x 7165126 10.1111/j.1439-0272.1982.tb02306.x

[CR45] Schneider CA, Rasband WS, Eliceiri KW (2012) NIH Image to ImageJ: 25 years of image analysis. Nat Methods 9(7):671–675. 10.1038/nmeth.2089 22930834 10.1038/nmeth.2089 PMC5554542

[CR47] Shahat AM, Rizzuto I, Klonisch T, Abdelrazek HMA (2018) Amelioration of diabetes-induced testicular and sperm dysfunction by taurine. Andrology 6(5):654–662. 10.1111/andr.12511 29978951 10.1111/andr.12511

[CR48] Shrilatha MB (2007) Early oxidative stress in testis and epididymal sperm in streptozotocin-induced diabetic mice: its progression and genotoxic consequences. Reprod Toxicol 23:578–587. 10.1016/j.reprotox.2007.02.001 17360155 10.1016/j.reprotox.2007.02.001

[CR49] Silva SFMD, Silva CHS, Dias FCR, Cordero-Schmidt E, Vargas-Mena JC, Silva IGMD, Báo SD, Carvalho TGD, Júnior RFDA, Moura CEBD, Melo FCSAD, Matta SLPD, Morais DB (2019) Testicular characterization and spermatogenesis of the hematophagous bat *Diphylla ecaudata*. PLoS ONE 14(12):e0226558. 10.1371/journal.pone.0226558 31835274 10.1371/journal.pone.0226558 PMC6910855

[CR50] Solis MY, Artioli GG, Gualano B (2021) Potential of creatine in glucose management and diabetes. Nutrients 13(2):570. 10.3390/nu13020570 33572228 10.3390/nu13020570 PMC7915263

[CR51] Taylor CR, Levenson RM (2006) Quantification of immunohistochemistry--issues concerning methods, utility and semiquantitative assessment II. Histopathology 49(4):411–424. 10.1111/j.1365-2559.2006.02513.x 16978205 10.1111/j.1365-2559.2006.02513.x

[CR53] Vandenberghe K, Goris M, Van Hecke P, Leemputte VM, Vangerven L, Hespel P (1997) Long-term creatine intake is beneficial to muscle performance during resistance training. J Appl Physiol 83(6):2055–2063. 10.1152/jappl.1997.83.6.2055 9390981 10.1152/jappl.1997.83.6.2055

[CR52] Van der Merwe J, Brooks NE, Myburgh KH (2009) Three weeks of creatine monohydrate supplementation affects dihydrotestosterone to testosterone ratio in college-aged rugby players. Clin J Sport Med 19(5):399–404. 10.1097/JSM.0b013e3181b8b52f 19741313 10.1097/JSM.0b013e3181b8b52f

[CR55] Wagner IV, Klöting N, Savchuk I, Eifler L, Kulle A, Kralisch-Jäcklein S, Dötsch J, Hiort O, Svechnikov K, Söder O (2021) Diabetes Type 1 negatively influences Leydig cell function in rats, which is partially reversible by insulin treatment. Endocrinology 162(4):bqab017. 10.1210/endocr/bqab017 33507237 10.1210/endocr/bqab017

[CR56] Zakir M, Ahuja N, Surksha MA, Sachdev R, Kalariya Y (2023) Cardiovascular complications of diabetes: from microvascular to macrovascular pathways. Cureus 15(9):e45835. 10.7759/cureus.45835 37881393 10.7759/cureus.45835 PMC10594042

[CR58] Zhang W, Tong L, Jin B, Sun D (2025) Diabetic testicular dysfunction and spermatogenesis impairment: mechanisms and therapeutic prospects. 10.3389/fendo.2025.1653975 10.3389/fendo.2025.1653975 PMC12414793 40927290

[CR57] Zirkin BR, Papadopoulos V (2018) Leydig cells: beyond testosterone. Biol Reprod 99(1):101–111 29566165 10.1093/biolre/ioy059 PMC6044347

